# Improved batch correction in untargeted MS-based metabolomics

**DOI:** 10.1007/s11306-016-1015-8

**Published:** 2016-03-18

**Authors:** Ron Wehrens, Jos. A. Hageman, Fred van Eeuwijk, Rik Kooke, Pádraic J. Flood, Erik Wijnker, Joost J. B. Keurentjes, Arjen Lommen, Henriëtte D. L. M. van Eekelen, Robert D. Hall, Roland Mumm, Ric C. H. de Vos

**Affiliations:** Biometris, Wageningen UR, Wageningen, The Netherlands; Laboratory of Genetics, Wageningen UR, Wageningen, The Netherlands; Horticulture and Production Physiology, Wageningen UR, Wageningen, The Netherlands; RIKILT, Wageningen UR, Wageningen, The Netherlands; Bioscience, Wageningen UR, Wageningen, The Netherlands; Laboratory of Plant Physiology, Wageningen UR, Wageningen, The Netherlands; Max Planck Institute For Plant Breeding Research, Cologne, Germany; Developmental Biology, Hamburg University, Hamburg, Germany

**Keywords:** Batch correction, Untargeted metabolomics, Non-detects, Mass spectrometry, *Arabidopsis thaliana*

## Abstract

**Introduction:**

Batch effects in large untargeted metabolomics experiments are almost unavoidable, especially when sensitive detection techniques like mass spectrometry (MS) are employed. In order to obtain peak intensities that are comparable across all batches, corrections need to be performed. Since non-detects, i.e., signals with an intensity too low to be detected with certainty, are common in metabolomics studies, the batch correction methods need to take these into account.

**Objectives:**

This paper aims to compare several batch correction methods, and investigates the effect of different strategies for handling non-detects.

**Methods:**

Batch correction methods usually consist of regression models, possibly also accounting for trends within batches. To fit these models quality control samples (QCs), injected at regular intervals, can be used. Also study samples can be used, provided that the injection order is properly randomized. Normalization methods, not using information on batch labels or injection order, can correct for batch effects as well. Introducing two easy-to-use quality criteria, we assess the merits of these batch correction strategies using three large LC–MS and GC–MS data sets of samples from *Arabidopsis thaliana*.

**Results:**

The three data sets have very different characteristics, leading to clearly distinct behaviour of the batch correction strategies studied. Explicit inclusion of information on batch and injection order in general leads to very good corrections; when enough QCs are available, also general normalization approaches perform well. Several approaches are shown to be able to handle non-detects—replacing them with very small numbers such as zero seems the worst of the approaches considered.

**Conclusion:**

The use of quality control samples for batch correction leads to good results when enough QCs are available. If an experiment is properly set up, batch correction using the study samples usually leads to a similar high-quality correction, but has the advantage that more metabolites are corrected. The strategy for handling non-detects is important: choosing small values like zero can lead to suboptimal batch corrections.

## Introduction

Mass spectrometry (MS) is the dominant detection technique in untargeted metabolomics experiments due to its sensitivity and information content. In many cases it also allows tentative annotations of metabolites on the basis of their observed accurate masses and mass spectra (de Vos et al. 2007; Patti et al. 2012; Dunn et al. 2013; Franceschi et al. 2014). Samples in metabolomics studies typically consist of complex matrices containing a large number of metabolites. Therefore, MS instruments are coupled to advanced chromatographic separation techniques including gas or liquid chromatography, or capillary electrophoresis. However, MS instruments need specialized operators, and chromatography and/or ionization of compounds are sensitive to external influences. As a result, it is virtually impossible to obtain exactly the same results in experiments repeated in different labs, on different machines, or even on the same machine during large series of samples taking several days for analysis. In particular batch-to-batch variation is commonly seen, where a batch is defined as a set of samples that have been extracted as well as measured in one uninterrupted sequence.

The goal of batch correction, then, is to remove these between-batch and within-batch effects, so that measurements across all batches are directly comparable. Batch variation can be dealt with in different ways, e.g., by using internal standards as controls, or by injecting reference or quality control samples (QCs) at regular intervals (Dunn et al. 2011; Hendriks et al. 2011). Spiking with internal standards has the disadvantage of potentially changing the physical sample, and since with untargeted experiments it is usually unknown in advance what compounds are going to be detected, there is the risk of using internal standards that coelute with metabolites of interest. Moreover, the added standards may not be representative for the specific chemical characteristics of the unknowns, and response factors may differ. As a result, this spiking approach is usually avoided in untargeted metabolomics. In contrast, including QCs for the entire technical procedure is common practice. Usually, a pooled sample comprising all or most study samples is used, so that the matrix characteristics of the QCs are similar to these real samples. Choosing the optimal number of QCs is not straightforward, as it depends on the type of material to be analyzed, the extraction procedure, the stability of the compounds in the extract, and finally the stability of the analytical system: injecting too many QCs leads to even longer sample series, and possibly more batches; injecting too few could make post-hoc corrections unfeasible. Applications ranging from injecting a QC every 4 up to 15 samples have been suggested (de Vos et al. 2007; Dunn et al. 2011; Kamleh et al. 2012).

A phenomenon that is often observed in metabolomics is the non-detect: a chemical feature found in some samples but completely absent in others, or (equivalently) perhaps present but at levels too low to be measured reliably. Non-detects will occur both at the level of the individual mass peaks and at the levels of metabolites. Another potential cause for non-detects is given by problems in data processing, e.g., leading to misalignments. We have taken utmost care to avoid this, and therefore we assume that this constitutes only a small minority of cases: non-detects therefore are assumed to correspond to low-intensity signals. Most data processing packages for MS-based metabolomics data use a threshold value (based on intensity, local signal-to-noise ratio or another characteristic) to define whether a feature is present in one particular sample or not. The resulting data table may contain many of these non-detects, sometimes simply represented by zeros, sometimes with a non-detect code.

For statistical analysis, it must be decided how to handle these non-detects. The data are left-censored: the intensity of non-detects is below a certain threshold, maybe even zero, but the exact value is unknown. Such information can be used, and several strategies to handle these non-detects exist. In most cases, one simply replaces these non-detects by a single value, e.g., zero, the limit of detection (LOD), or a number in between these two possibilities (Hughes et al. 2014; Xia et al. 2015). A more elaborate approach is to use multiple imputation (Little and Rubin 1987; Schafer 1996), basically a repeated replacement of non-detects with random numbers from a predefined distribution. Although the analysis then becomes more complicated and computer-intensive, results have been shown to be quite good (Uh et al. 2008). The objective of this paper is to obtain adequately corrected values for the data that have been measured rather than to obtain a completed data table, and therefore we are not considering multiple-imputation approaches here. Finally, a baseline-type of approach for handling non-detects is simply to ignore them, and to base the correction only on those values that are detected. The disadvantage is that potentially valuable information (non-detects representing small numbers below a threshold) is lost.

This paper describes a systematic analysis of different strategies to perform batch correction in the presence of non-detects. Both strategies requiring the presence of QCs and more generally applicable strategies are investigated, as are the benefits of explicitly including batch and injection sequence information. The concepts are illustrated using three data sets from different untargeted metabolomics platforms for measuring *Arabidopsis* samples, i.e., GC–MS for detecting volatiles, GC-ToF-MS for derivatized polar extracts, and accurate-mass UPLC–MS for semi-polar compounds. For the evaluation of the different strategies, we propose two quality criteria: one is based on principal component analysis [PCA, (Jackson 1991; Jolliffe 1986)], and the other on the variation within biological replicates.

## Batch correction

Several different algorithms are available to perform batch correction [see, e.g., Fernández-Albert et al. (2014)]. Rather than do an exhaustive comparison of different approaches, we focus on the amount of information provided to the methods, and we consider two generic cases:

- explicitly taking into account batch information and, possibly, injection sequence information. For this approach QCs can be used but are not required;
- correction without explicit batch or injection sequence information. QCs are mandatory in this case.

### Batch correction using all available information

When both batch and injection sequence information are used, batch correction is usually done metabolite-wise in an Analysis of Covariance (ANCOVA) framework (Hendriks et al. 2011; Kirwan et al. 2013):

$$\begin{aligned} x_{c, i} = x_{u, i} - \hat{x}_i + \bar{x} \end{aligned}$$

where $x_{c, i}$ and $x_{u, i}$ are the corrected and uncorrected intensities for metabolite *x* in injection *i*, respectively, and $\bar{x}$ is the average intensity of this metabolite across all batches. The predicted intensities $\hat{x}_i$ in this example can be obtained by linear regression. If injection order information $S_i$ is available, this can be used next to the information on batch labels $B_i$:

$$\begin{aligned} \hat{x}_i = a S_i + b B_i + \epsilon \end{aligned}$$

where *a* and *b* are coefficients to be determined. If no order information is available, this reduces to

$$\begin{aligned} \hat{x}_i = b B_i + \epsilon. \end{aligned}$$

The safest option is to fit these “correction models” using the QCs: there, one can be sure that the true underlying value is constant, and that one should measure the same intensity in all batches and for all parts of the injection sequence within a batch. When too few QCs are available to do this reliably (which can easily happen for less abundant metabolites, even when the number of QCs itself is large enough) these predictions can also be based on the study samples, provided that these are properly randomized. The assumption then is that there is no relation between injection order within a batch and intensity, and between batches (Dunn et al. 2011). In the following, correction strategies based on QCs will be referred to with the letter Q; strategies based on the study samples with the letter S.

Non-detects can severely disturb the estimation of the correction terms. Here, we compare several approaches to estimate the batch and injection order effects. For strategies based on the QCs (Q), this leads to the following variants:

Q Simply ignore the non-detects, and use linear regression to fit the correction lines using only the detected values.
Q0 Impute the non-detects by a value of zero. Although this is an often-used approach, a possible danger is that this value is too extreme and may lead to poor corrections.
Q1 Impute the non-detects by a value that is half the detection limit; one could argue that the real value is somewhere between zero and the detection limit, and in the absence of any other information, half of the detection limit would be the most logical estimate (Xia et al. 2015).
Q2 Impute by the detection limit itself. Usually, no detection limit is known, but often the smallest value present in the data set is taken as a reasonable estimate.
Qc Use censored regression rather than least-squares regression without imputation. In censored regression, information is used that the non-detects are below a certain limit, without knowing their exact value. The choice of this limit is important: knowing that a certain value is below, e.g., 10,000 gives different information than knowing that it is below 10. In this paper, tobit regression (Greene 2003; Tobin 1958) was used with left-censoring at the smallest value found in the data set (taken as LOD).

Thus, five different ways of handling non-detects in both Q and S strategies are considered, ten methods overall. Note that these strategies are all univariate: the corrections are done for every metabolite separately.

### Normalization approaches

Normalization approaches do not explicitly correct for batch and injection order effects but rather utilize the fact that QCs are technical replicates: their intensities should be independent of batch label or injection number (Draisma et al. 2010; Veselkov et al. 2011; Hughes et al. 2014). An interesting example of such a strategy is the identification and subsequent removal of unknown structured variation on the basis of control samples in an RNASeq context (Risso et al. 2014), an extension of earlier work on microarray experiments (Gagnon-Bartsch and Speed 2012). Recently, this “Removal of Unwanted Variation” (RUV) strategy has also been applied to metabolomics data (Livera et al. 2015). The method is based on modeling the subspace of the unwanted variation, by performing a PCA on the data of the QCs. The projection of all study samples in this subspace gives an estimate of the unwanted variation for these samples, which can subsequently be removed. In contrast to the approaches mentioned above, RUV is a multivariate method. It has one control parameter *k*, the number of principal components (PCs) defining the subspace of unwanted variation. In this paper, we use a value of $k = 3$; very similar results are obtained for values in the range of 3–10 (data not shown). Missing values are not allowed in this method, so we again impute non-detects by the same three levels used in the Q and S strategies, leading to methods R0, R1 and R2.

The total set of evaluated methods is summarized in Table 1.

**Table 1:** Overview of batch correction methods considered in this paper

Method	Based on	Non-detects	Methodology
Q	QCs	NA	LS regression
Qc	QCs	NA	Censored regression
Q0	QCs	0	LS regression
Q1	QCs	LOD/2	LS regression
Q2	QCs	LOD	LS regression
S	Study	NA	LS regression
Sc	Study	NA	Censored regression
S0	Study	0	LS regression
S1	Study	LOD/2	LS regression
S2	Study	LOD	LS regression
R0	QCs	0	PCA
R1	QCs	LOD/2	PCA
R2	QCs	LOD	PCA

Methods “Q” are based on different forms of regression using the QCs, methods “S” on regressions using the study samples, and “R” on the RUV method, a PCA of the QCs. Non-detects are handled as missing values (NA) or imputed with a single value (0, LOD/2, or LOD), column “non-detects”

### Evaluation of batch corrections

Two quality criteria have been designed and tested to assess the success of a particular batch correction:

1. The first approach is based on PCA. Score plots often provide a simple and easily interpretable visual check of the presence of batch effects. As a quantitative criterion, we proposes to use the average distance between batches, based on their scores. As a distance measure between two batches we use the Bhattacharyya distance, basically the distance between two normally distributed point clouds:
  $$\begin{aligned} D_B = \frac{1}{8} (\mu _1 - \mu _2)^T \varSigma ^{-1} (\mu _1 - \mu _2) + \frac{1}{2} \left( \frac{\det \varSigma }{\sqrt{\det \varSigma _1 \det \varSigma _2}} \right) \end{aligned}$$
  where $\mu _1$, $\mu _2$, $\varSigma _1$ and $\varSigma _2$ are the means and covariance matrices of the two distributions, in this case the PCA scores of the two batches, and
  $$\begin{aligned} \varSigma = \frac{\varSigma _1 + \varSigma _2}{2} \ . \end{aligned}$$
  The smaller this average Bhattacharyya distance, the larger the overlap between the batches and the smaller the batch effects. In this paper have used two PCs for calculating the PCA criterion (also because of the visualization possibilities) but, in our experience, the conclusions do not critically depend on this choice. Again, for calculating the PCA scores no non-detects are allowed: to avoid any influence of different numbers of non-detects in the individual correction strategies, in this quality criterion non-detects are imputed by column (metabolite) averages, so that they will be zero after scaling and do not influence the results of the criterion. To avoid highly abundant metabolites to dominate the criterion, the columns of the data matrix (metabolites) are standardized to mean zero and unit variance before calculating the QC value.
2. The second approach is based on the presence of biological replicates. The variation within one group (here: a genotype) consists of biological variation and technical variation. Batch correction should decrease the latter, so after correction the within-genotype variation is expected to be smaller than before correction. This can be measured by calculating, for each individual metabolite, the fraction of variance accounted for by the biological variation, also known as the repeatability:
  $$\begin{aligned} \text{ repeatability } = \frac{\hat{\sigma }^2_{\tiny \mathrm{between}}}{\hat{\sigma }^2_{\tiny \mathrm{between}} + \hat{\sigma }^2_{\tiny \mathrm{within}}} \approx \frac{\hat{\sigma }^2_{\tiny \mathrm{biol}}}{\hat{\sigma }^2_{\tiny \mathrm{total}}}\ . \end{aligned}$$
  The within-group variance $\hat{\sigma }^2_{\tiny \mathrm{within}}$ is given by the pooled variance over all groups (genotypes); the between-group variance $\hat{\sigma }^2_{\tiny \mathrm{between}}$ is the variance between the group means. This formulation by definition leads to a number between zero and one, independent of the measurement scale. Averaging over all metabolites gives an overall repeatability estimate. Similar measures have been used in literature before [(see, e.g., Trutschel et al. (2015)].

In both cases the quality criteria are based on the study samples only: QCs are not considered.

## Materials and methods

### Data

The performance of all correction methods in the previous section was assessed by applying them to three different data sets of *Arabidopsis* samples. These differ in sample analysis characteristics such as batch length, number of QCs per batch, and the number of biological replicates, allowing for a thorough evaluation of the strong and weak points of the correction methods. It should be noted that in each of these cases utmost care has been taken to avoid batch effects. Nevertheless, as also has been noted before (Dunn et al. 2011; Hendriks et al. 2011), they cannot always be avoided, and have to be dealt with.

Each of the three experiments described below was performed with one single column, with no other types of samples measured in between, in one consecutive time block. Given that a single MS analysis would take between 30 and 60 min, the measurement time was $\sim$1 week for data set III, and more than 2 weeks for data sets I and II.

In all cases, variables are relative intensities associated with reconstructed metabolites, defined as a group of mass features most likely originating from the same metabolite. The values given for each reconstructed metabolite corresponds to the total ion count of a chromatographic peak and therefore does not represent a single mass feature only.

#### Set I: LC–MS data of a large Arabidopsis hapmap population

Seeds from 357 natural accessions of *Arabidopsis*, collected worldwide (Li et al. 2010; Horton et al. 2012), were sown on filter paper with demi water and stratified at 4 $^\circ$C in dark conditions for 5 days. Subsequently, seeds were transferred to a culture room (16 h LD, 24 $^\circ$C) to induce seed germination for 42 h. Six replicates per accession were transplanted to wet Rockwool blocks of 4 $\times$ 4 cm$^2$ in a climate chamber (16 h LD, 125 $\upmu{\text{ mol }} /{\text{m}}^2{\text{s}}$, 70 % RH, 20/18 $^\circ$C day/night cycle). All plants were watered daily for 5 min with 1/1000 Hyponex solution (Hyponex, Osaka, Japan). Plants were harvested 29 days after germination and leaves of three plants were pooled in two replicate samples each. Samples were ground in liquid nitrogen and an aliquot of all samples was mixed to generate the large pool needed for preparing the QCs. These were independently and simultaneously weighed and extracted with the study samples (5–6 times per batch) and injected at regular intervals within the analysis series. In total, 51 QCs were injected. Batch sizes ranged from 78 to 80 samples, with the exception of the last batch, batch 10, containing 48 samples.

For the LC–MS analysis, aqueous-methanol extracts were prepared from 50 mg frozen ground material to which 200 $\upmu$l of 94 % MeOH containing 0.125 % formic acid was added (de Vos et al. 2007). After sonication and filtering, the crude extracts were analyzed as described previously (van Duynhoven et al. 2014) using UPLC (Waters Aquity) coupled to a high-resolution Orbitrap FTMS (Thermo). A 20 min gradient of 5–35 % acetonitril, acidified with 0.1 % formic acid, at a flow rate of 400 $\upmu$l/min was used to separate compounds on a 2.1 x 150 mm$^2$ C18-BEH column (1.7 $\upmu$m particle size) at 40 $^\circ$C. Metabolites were detected using a LTQ-Orbitrap hybrid MS system operating in negative electrospray ionization mode heated at 300 $^\circ$C with a source voltage of 4.5 kV [more details are described in van Duynhoven et al. (2014)]. The transfer tube in the ion source was replaced and the FTMS recalibrated after each sample batch, without stopping the UPLC system.

After preprocessing, metabolites occurring in fewer than 20 different genotypes were removed, leading to a data matrix containing relative intensities of 567 reconstructed metabolites in 761 samples (including the QCs). The percentage of non-detects in this matrix is 48 %. For individual metabolites, the fraction of non-detects can be much larger, and in this data set is up to 97 %.

#### Set II: GC–MS of volatiles of the Arabidopsis hapmap population

This dataset is based on aliquots of the same *Arabidopsis* material as described for data set I. The aim here was to analyse volatile organic compounds (VOCs) present in the leaf material using solid phase microextraction (SPME) of the headspace. Extracts of 50 mg from frozen ground material treated as described by Verhoeven et al. (2012) and Mumm et al. (2015) were analysed on a GC–MS system (Agilent GC7890A with a quadrupole MSD Agilent 5978C) as described by Cordovez et al. (2015). In contrast to the aforementioned study, the temperature program of the GC oven started at 45 $^\circ$C (2 min hold) and rose first with 8–190 $^\circ$C min$^{-1}$, followed by 25–280 $^\circ$C (2 min hold). This data set contains information on 753 injections (including QCs) with, in total, 40 % non-detects, similar to what was found in the LC–MS data. For individual metabolites, the percentage of non-detects goes up to 97 %.

Again, only those metabolites were retained that were present in at least 20 different genotypes, in this case 603 metabolites. Fifteen batches of 34–99 samples were used, with on average 15 study samples per QC; the total number of QCs is 50.

#### Set III: GC-ToF-MS polar metabolite data of an Arabidopsis nucleotype-plasmotype diallel study

This dataset is based on the analysis of polar extracts from a nucleotype-plasmotype combination study of *Arabidopsis* for 58 different genotypes. For details of the used plant material we refer to Flood (2015). Analysis of the polar, derivatized metabolites by GC-ToF-MS (Agilent 6890 GC coupled to a Leco Pegasus III MS) and processing of the data were done as described in Villafort Carvalho et al. (2015). Here, the number of metabolites (75) is much lower than in the other two data sets, partly because the focus was on the primary rather than the secondary metabolites. The number of samples was 240, with a percentage of non-detects of 16 %; the maximum fraction of non-detects in individual metabolites is 92 %. All metabolites were retained in the analysis. Four batches of 31–89 samples were employed, containing 2–6 QCs per batch, 14 in total. Four biological replicates were present for each accession, but unlike the previous two data sets these biological replicates are not spread evenly over the batches.

### Software

Processing of the data was performed using the Metalign (Lommen 2009) (for extracting and aligning mass features) and MSClust (Tikunov et al. 2012) (for clustering mass features on the basis of their similarities in both retention time and abundance patterns across samples) according to a pipeline described in more detail elsewhere (Lopez-Sanchez et al. 2015; Roldan et al. 2014). All further calculations were performed in R (R Core Team 2015), version 3.2.3, using packages **AER** for tobit regression (Kleiber and Zeileis 2008), **fpc** for the Bhattacharyya distance (Hennig 2014), **ChemometricsWithR** for PCA (Wehrens 2011), and **RUVSeq** for the RUV method (Risso et al. 2014). The latter is available from the Bioconductor repository^1^; all others are available from CRAN.^2^ Further functions for batch correction and evaluation of batch effect sizes were written in-house. These functions, as well as anonymized versions of the data sets, are available in the form of an R package, so that all results in this paper can be reproduced exactly. It can be installed directly from https://github.com/rwehrens/BatchCorrMetabolomics.

## Results and discussion

Below, the results of the different forms of batch correction are compared for the three data sets, addressing issues such as the handling of non-detects. In particular, it has been investigated how much the explicit inclusion of batch labels and injection order improves the correction, and how important the presence of QC information is in this respect. When a correction is not possible for a particular metabolite in a sample, the original uncorrected value is retained in the corrected matrix, so that the evaluation of the results is always done on the basis of an equal number of data points. We will come back to this in the last part of the results section.

### Set I: LC–MS data of the *Arabidopsis* hapmap population

**Fig. 1:**
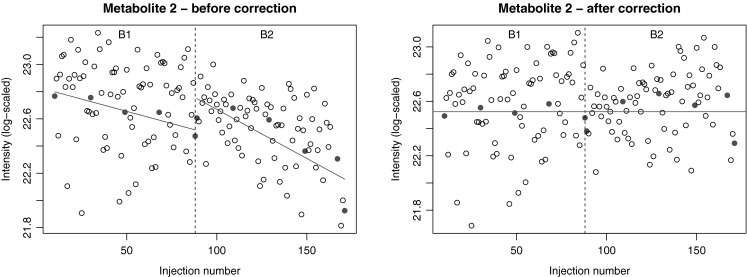
Data for a single metabolite measured in two batches of $\sim$80 samples each. **a** Showing uncorrected data, there is a clear overall intensity difference between the batches, and a gradual intensity decrease within both batches. QCs are indicated by *red dots*, study samples with *circles*. Correction lines fitted through the QCs in the individual batches are indicated by the *red lines*. The intensities after correction are shown in **b**

Partly due to a particularly unfortunate series of events including a broken oil pump and multiple power cuts, data set I shows substantial batch effects. Figure 1a, b depicts data from one particular metabolite in the first two batches of the LC–MS data. Clearly, apart from the global intensity differences between the batches, a trend within each batch can be observed. The correction lines estimated using the QCs are indicated; these lines are basically subtracted from the measurements, so that the corrected intensities shown in the right panel are directly comparable across batches. Since in this set the number of QCs is large enough and injection order clearly is important, for this data set only forms of strategies Q and S taking into account also the injection order were used.

**Fig. 2:**
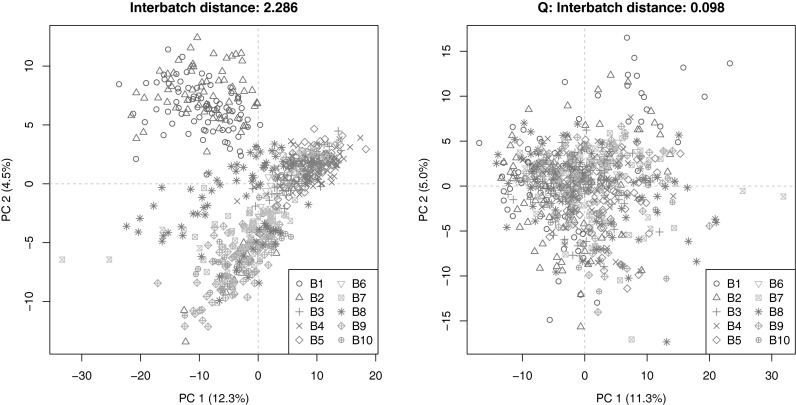
PCA plots of the LC–MS data for the *Arabidopsis* hapmap population (data set I). **a** Shows the uncorrected data where the different batches can clearly be recognized, especially batches 1 and 2. **b** Shows, as an example of what can be achieved, the result after correction with strategy Q

In Fig. 2a, the PCA scores for the individual, uncorrected, samples are shown with different symbols and colours to indicate the batch labels. The average inter-batch distance in this PCA space is 2.286. As an example of what can be achieved, Fig. 2b shows the PCA scores after correction using strategy Q (based on the QCs, not using imputed values for the non-detects). No obvious batch effects are visible anymore. Also the much lower value of the PCA criterion shows that the differences between the corrected batches have all but disappeared.

**Fig. 3:**
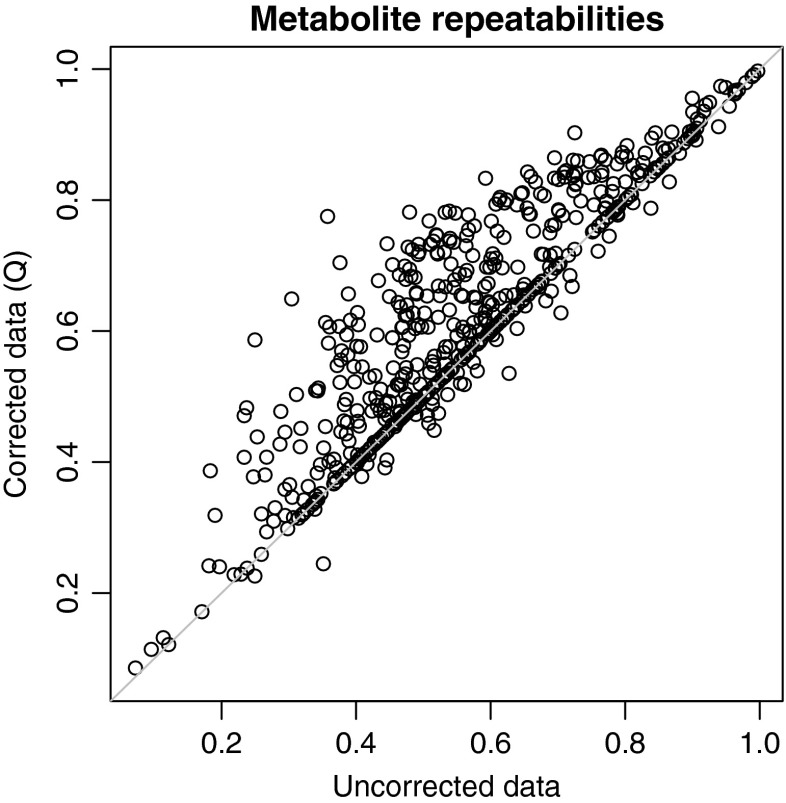
Repeatabilities for individual metabolites. Uncorrected data on the *x* axis; corrected data (strategy Q) on the *y* axis. In almost all cases repeatabilities show an improvement upon correction

Similarly, Fig. 3 shows that the repeatabilities for virtually all metabolites improve upon correction by strategy Q, leading to an increase in the average repeatability from 0.559 to 0.62. A certain number of metabolites cannot be corrected because not enough information is present in the QCs: these are lying on the diagonal of the plot.

**Fig. 4:**
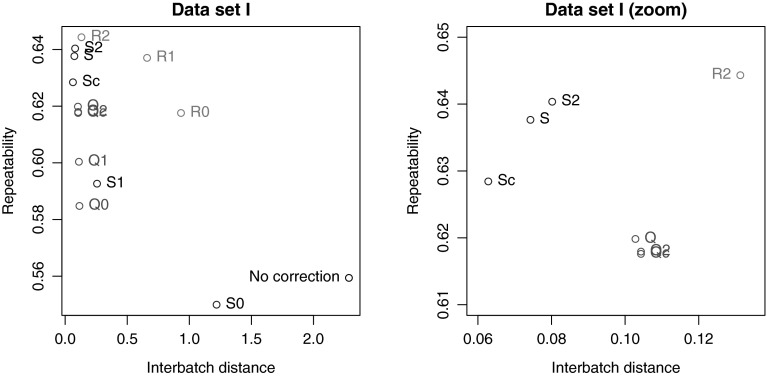
Comparison of the performance of the batch correction methods for the LC–MS *Arabidopsis* hapmap data set. The best values are in the* top left corner*: low values for the PCA distance criterion on the *x* axis, and high repeatabilities (*y* axis)

The comparison between the different batch correction strategies for this data set is shown in Fig. 4. The best methods are those with a small value for the interbatch distance and a high repeatability, *i.e.*, points in the top left corner of the figures. Clearly, virtually all correction methods considered lead to substantial improvements in both quality criteria in comparison to the uncorrected data. The best results are obtained when the LOD value is used to replace non-detects; imputing with zero or half the LOD value leads to clearly inferior results. This data set in a way provides the ideal case for batch correction: it has relatively large batches of more or less equal size, and a sufficiently high number of QCs. Indeed, zooming in on the optimal region (the right plot in Fig. 4) shows that all three strategies (Q, R and S) have representatives in this area, indicating that whatever the strategy chosen it is possible to obtain a good result. Still, the Q strategies are dominated by the S and R strategies. The performance of the R2 method is especially impressive, since it is not provided with batch and injection order information that is available to the other methods. Of course, the fact that it is a multivariate method does allow to borrow strength across metabolites, and in addition the method in principle is able to correct for any unknown structured variation.

### Set II: GC–MS data of the hapmap population

**Fig. 5:**
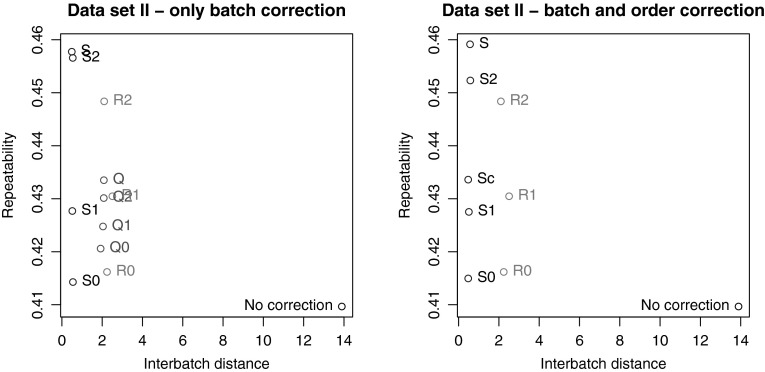
Results of the corrections for the hapmap GC–MS data. **a** Corrections based on batch information only (strategies Q and S). **b** Batch information as well as injection sequence are used in the correction with the S strategies. The values for the RUV corrections and the uncorrected data are the same in both panels

The *Arabidopsis* hapmap population was also analysed using GC–MS. Here, batch effects were to be expected because of airconditioning breakdown during the measurements. Shorter batches were used, resulting in fewer QCs per batch. Therefore, it is impossible to use strategy Q for correcting both batch effects and injection order effects: the correction lines cannot be estimated reliably. For Q strategies, only a correction using batch information has been performed. Since the number of study samples is much larger than the number of QCs, it is possible to use strategy S compensating only for batch effects and for within-batch drift.

The results are shown in Fig. 5. The left panel contains the results of the different batch corrections where no within-batch drift is taken into account. All methods lead to considerable improvements in the interbatch distance (the *x*-axis) over the uncorrected data. Again, imputation with zero or half the LOD is suboptimal. The best results here are obtained with strategy S, simply ignoring the non-detects. In particular, this clearly beats the Q strategies. In Fig. 5b injection order within batches is taken into account for the S strategies. As discussed before, Q strategies are not applicable because of a lack of QCs. The results for the S strategies are virtually the same in both panels: for this data set, injection order does not seem to be an important factor.

### Set III: GC-ToF-MS data for the diallel study

**Fig. 6:**
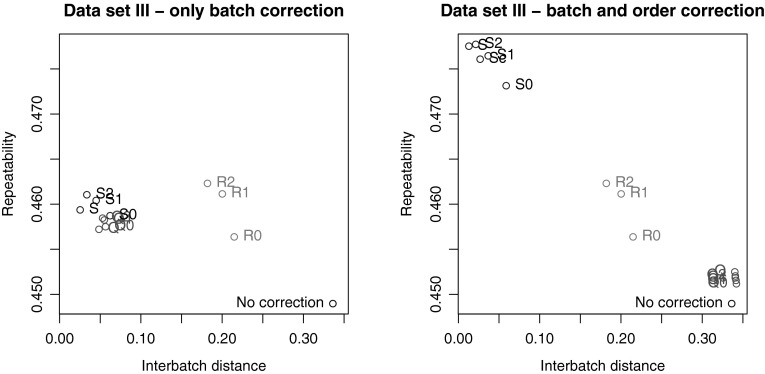
Correction results for the diallel study data set. **a** Corrections based only on batch averages; **b** corrections based on batch and injection order information. In both panels the points for the RUV corrections and uncorrected data are identical

The third data set is characterized by a relatively low number of metabolites and a smaller fraction of non-detects, compared to the other two sets. Figure 6a shows the results of batch correction when within-batch drift is not taken into account. Clearly, the batch effects to begin with are much smaller than in the other data sets (compare the value of the PCA criterion for the uncorrected data with the values in Figs. 4 and 5). The influence of the non-detects is also much smaller: the three strategies lead to clearly distinguished clusters, and only in the R strategies any effect of different imputations is visible.

 Figure 6b shows the results when injection order is taken into account in the correction. The improvement in the repeatability results for strategy S is striking: here, the S correction models clearly outperform the other correction methods. In contrast, the Q strategies perform worse than in the situation where injection order is ignored. The reason for this behaviour lies the small number of metabolites for which such a correction is possible: a large part remains uncorrected, and therefore the results are close to the original data. The next section quantifies this in more detail. Overall, this data set shows an example where including batch and injection order information is essential for arriving at an optimal correction, and where it is better to rely on the study samples rather than the QCs. Probably because of the low number of QCs, also RUV is not able to arrive at the same quality level.

### Extent of the corrections

Regression-based batch correction such as strategies Q and S are univariate methods, appliccable when for a particular metabolite sufficient information is present to estimate the correction lines. This is not always the case. In pooled samples, for example, metabolites that are present in only a minority of the samples may be present in such low amounts that they cannot be detected, and as a consequence batch correction based on the QCs is unreliable. Also when using the study samples it may happen that a metabolite is detected in too few cases. For a particular metabolite these issues may show up in some batches only, allowing a correction of the batches for which enough information is available and leaving the other batches uncorrected. When batch correction is performed without taking into account injection order, this effect is less pronounced since averages can be calculated with fewer samples than correction lines can.

In Table 2 an overview is given for the three data sets of the number of metabolite/batch combinations for which a correction has proved impossible. The differences between strategies Q and S are clear: the number of uncorrected cases in Q strategies (depending on QCs) is much higher than in S strategies (depending on study samples). Similarly, using injection order in strategies Q and S leads to a drastic decrease in the number of cases for which a correction is possible. In particular for the correction of data set III with Q strategies there are many cases for which such a correction is impossible, due to the fact that in only two out of four batches at least four QCs were present. If, instead of replacing values in the original matrix with corrected values, we would evaluate only the corrections, then we would see that the corrections themselves would lead to very good values for the two quality criteria. However, plots like Fig. 6 would be very hard to interpret, since for each class of correction methods different numbers of metabolites would be taken into account.

The one big advantage of the RUV normalization approach, not relying on batch-wise correction estimates, is that all detected metabolites will be corrected. That is not to say that all metabolites play a part in determining the correction: if a particular metabolite is not present in the QCs it will not contribute to the definition of the PC space covering the unwanted variation.

**Table 2:** The percentage of cases (metabolite/batch combinations) for which correction is impossible for the three data sets and the correction strategies considered

	Data set I (%)	Data set II (%)	Data set III (%)
Q (ave)	–	29.2	14.3
Q (lin)	37.1	–	58.0
S (ave)	–	5.6	1.3
S (lin)	9.0	11.3	2.3
R	0.0	0.0	0.0

Injection order is not taken into account in the lines denoted “ave”; it is in the lines denoted “lin”

## Conclusion

This paper addresses the important topic of batch correction in untargeted MS-based metabolomics experiments. Using three large data sets, measured on different instruments, and containing repeated measurements of one pooled QC sample as well as measurements of biological replicates, it was possible to investigate the performance of several commonly used batch correction methods. A clear picture has emerged. If many QCs are present within batches, they can be used to good effect for correcting both between-batch and within-batch effects. Especially for longer batches the injection order within a batch can have a large influence on the results as well, and can be corrected for by explicitly including this information in the correction method. Corrections can not only be based on the QCs, but also on the study samples themselves – in the optimal situation with a reasonably large number of QCs, the results are mostly comparable. When the number of QCs is not very large, however, correction on the basis of the study samples may be the preferred option.

The corrections using the study samples have the advantage that they can be calculated for a larger number of metabolites. Corrections based on the QCs can only be done for those metabolites that are actually present in the QCs. The normalization method investigated in this paper, RUV, did not use batch or injection order information at all. This led to results that were comparable in quality to the other two strategies for the hapmap samples (both LC and GC), but led to inferior results in the last data set. The main advantage of the RUV method is that all measured values are corrected, whereas for the other correction methods the number of corrected metabolites was always smaller than the total number, sometimes quite substantially so. RUV is the only method of the ones considered here that is able to decrease the effects of other sources of technical variation like MS detector sensitivity and perhaps even ion suppression.

The situation of non-detects warrants careful investigation. For batch correction, at least, we have seen detrimental effects of replacing non-detects with small values like zero, or half the LOD. Using the smallest value in the data set (LOD) is better. Instead of imputing values, censored regression methods can be used to good effect, and one can even ignore the non-detects and base the corrections only on detected features. Also in that case the results are quite good, especially for the S strategies where the number of points is larger. We have also considered robust regression methods that are less sensitive to outliers, to see if the effect of a particularly unlucky choice of imputed value can be remedied. Indeed, when using, e.g., Huber’s M-estimators (Huber 1981) to calculate the correction lines, the results for strategies like S0 and Q0 improved quite significantly, but still they did not reach the same levels as the other strategies (data not shown). A disadvantage, especially for the Q0 and Q1 strategies is also the relatively low number of QCs: robust regression is not very useful when only four or five points are available for estimating the parameters of the correction line.

The two quality criteria introduced in this paper give an easy and quantifiable way to assess the success of batch correction. The PCA-based criterion using the Bhattacharyya distances between batches is generic and allows visual identification of samples, or groups of samples, that do not conform to the general trend. Here, we have restricted ourselves to a criterion based on the first two PCs, also because of our aim to visualize the results. In principle, one could also take higher-order PCs into account, but this in our experience did not lead to different conclusions. The second quality criterion is based on the presence of biological replicates, ideally measured in different batches. The definition, a fraction of variance explained, leads to numbers on a scale from zero to one, which can easily be interpreted. As with the PCA-based criterion, individual outliers can be investigated, leading to potentially valuable information.

The batch correction strategies described in this paper have been applied to relative metabolite intensities, but in principle they can also be used for correcting non-aggregated individual mass peaks. Since the correction itself is quite simple, the added computational complexity is not a major concern. However, we would still advise against this practice as any errors at the peak level that would be less influential on the level of the metabolite as a whole (e.g., misalignment of a single mass trace) can severely disturb the batch correction, thereby hampering subsequent data interpretation.

Batch correction based on the study samples assumes that the sample injection sequence has been properly randomized. It is shown that results can be very good. This finding could lead to a reassessment of the number of QCs required in long injection sequences: QCs serve other purposes, such as checking the efficiency of extraction, too, but in some cases their number could be decreased when they are no longer needed for batch correction.

## Footnotes

1 http://www.bioconductor.org

2 http://cran.r-project.org
